# Transcriptomic and physiological analyses of *Trichoderma citrinoviride* HT-1 assisted phytoremediation of Cd contaminated water by *Phragmites australis*

**DOI:** 10.1186/s12866-024-03252-1

**Published:** 2024-03-21

**Authors:** DaWei Chen, YiHan Wang, Ni Li, YaLi Huang, YiFan Mao, XiaoJun Liu, YaRong Du, Kun Sun

**Affiliations:** 1 College of Life Sciences, Northwest Normal University, Lanzhou, 730070 Gansu China; 2 Key Laboratory of Strategic Mineral Resources of the Upper Yellow River, Ministry of Natural Resources, Lanzhou, 730046 China

**Keywords:** Phytoremediation, Plant growth promoting microbe, Plant–microbe association, Cd contaminant, Mechanism

## Abstract

Plant growth promoting microbe assisted phytoremediation is considered a more effective approach to rehabilitation than the single use of plants, but underlying mechanism is still unclear. In this study, we combined transcriptomic and physiological methods to explore the mechanism of plant growth promoting microbe *Trichoderma citrinoviride* HT-1 assisted phytoremediation of Cd contaminated water by *Phragmites australis.* The results show that the strain HT-1 significantly promoted *P. australis* growth, increased the photosynthetic rate, enhanced antioxidant enzyme activities. The chlorophyll content and the activity of superoxide dismutase (SOD), peroxidase (POD), catalase (CAT) and ascorbate peroxidase (APX) were increased by 83.78%, 23.17%, 47.60%, 97.14% and 12.23% on average, and decreased the content of malondialdehyde (MDA) by 31.10%. At the same time, strain HT-1 improved the absorption and transport of Cd in *P. australis*, and the removal rate of Cd was increased by 7.56% on average. Transcriptome analysis showed that strain HT-1 induced significant up-regulated the expression of genes related to oxidative phosphorylation and ribosome pathways, and these upregulated genes promoted *P. australis* remediation efficiency and resistance to Cd stress. Our results provide a mechanistic understanding of plant growth promoting microbe assisted phytoremediation under Cd stress.

## Introduction

In recent decades, the improper disposal of industrial and domestic waste, the excessive use of agricultural chemicals and the unreasonable discharge of sewage from human activities, such as metal mining and smelting have caused various heavy metal pollutants to enter the water. This has resulted in the water pollution [1]. Among all heavy metals, cadmium (Cd) has been intensively studied due to its high mobility and solubility (bioavailability), as well as its strong cumulative toxicity [2, 3]. As a typical toxic heavy metal, Cd is one of the most harmful and ubiquitous water environmental pollutants [4], which has significant toxic effects on plants [5, 6]. At the same time, Cd is classified as a class I carcinogen, which poses huge health risks to the human body as it passes through the food chain [7, 8]. At present, the use of Cd in industry continues to increase, making it an escalating hazard factor [9]. Therefore, remediation of water contaminated with heavy metals, particularly Cd, is of great importance for ensuring environmental safety and human health, and is also very necessary and urgen.

Traditional Cd pollution control technologies include precipitation method, ion exchange method, membrane separation method, adsorption method, etc. [10]. However, these physical and chemical remediation methods often encounter issues such as low efficiency in remediation, high economic costs, and the potential for secondary pollution [11]. In the past decade, plant remediation has gradually become a trend due to its economic, aesthetic and environmental protection characteristics [12]. A previous study reported that *Pistia stratiotes* has shown promising remediation effects on water contaminated with Cd [13]. *Celosia argentea* Linn, *Populus deltoids* and *Salix viminalis* were found to be effective in remediating Cd-contaminated soil [14–16]. However, natural factors such as environment, climate and the stress of heavy metals on plants have limited the scope and effect of phytoremediation. Therefore, it is necessary to enhance the exogenous reinforcement methods in order to achieve the desired heavy metal remediation effect in the real environment. Studies have found that the interaction between plants and beneficial microorganisms, especially plant growth-promoting microbes (PGPM) can significantly improve the remediation efficiency of plants to heavy metal Cd [17–19]. Kamran et al. isolated a plant growth-promoting bacteria (PGPB) *Pseudomonas putida* from the heavy metal contaminated soil and found that bacterial inoculation increased biomass of Cd hyperaccumulator plant *Eruca sativa* by up to 33%, and Cd uptake increased by 29% [20]. Ma et al. isolated a PGPB *Achromobacter piechaudii* from the stem of hyperaccumulator *Sedum plumbizincicola*, which significantly increased the bioavailability of Cd, Zn and Pb, promote plant growth and enhanced plant uptake of Cd, Zn and Pb [21]. Wu et al. inoculated PGPB *Pseudomonas fluorescens* isolated from the stems of *Sedum alfredii* into plants, which significantly increased root biomass and Cd accumulation in plants, with differences of 1.82 times and 3.04 times compared with the control, respectively [22]. However, so far, the key processes and mechanisms of PGPM synergistic remediation of heavy metal pollution have not been fully understood, especially at the molecular level.

*Phragmites australis* is a perennial graminaceous plant with high biomass productivity [23]. It is widely distributed throughout the world and can survive in acidic conditions. In recent years, extensive research and application have found that compared with other wetland plants, *P. australis* is better at accumulation of some heavy metals [24]. Bragato et al. discovered that riparian wetland *P. australis* opens the root-leaf transport system at the end of the growing season, transferring heavy metals from the root system to the aging leaf tissue, and removing heavy metal toxicity from the body through leaf apoptosis [25]. Bernardini et al. found that under high concentrations of Zn and Pb in hydroponics, the physiological activity of photosynthetic organs in *P. australis* was inhibited, but the roots had a better ability to enrich heavy metals [26]. In summary, *P. australis* can be used to assess heavy metal pollution in wetlands and it is an excellent plant for mitigating heavy metal pollution in water.

*Trichoderma citrinoviride* HT-1 was isolated from the root of *Rheum palmatum* in the early stage of our research group. It has strong vitality and outstanding colonization ability. *T. citrinoviride* HT-1 can produce IAA and siderophores to promote plants growth, and has good inhibitory effect on many plant pathogens [27]. In addition, the Cd tolerance of strain HT-1 was determined, the results showed that strain HT-1 has good Cd tolerance. In this study, physiological and transcriptomic methods were used to explore the mechanism of *Trichoderma citrinoviride* HT-1 improve the tolerance and repair efficiency of *P. australis* to Cd. The purpose of this study is to (a) explore the mechanism of strain HT-1 enhances the Cd tolerance of *P. australis*; (b) the accumulation and transport of Cd in *P. australis* plants under the treatment of strain HT-1 were analyzed; (c) identify differentially expressed genes (DEGs) and their key pathways; and (d) reveal the molecular mechanism of plant growth-promoting microbes to improve the tolerance and repair ability of *P. australis* to heavy metals. This study will provide a strong scientific basis for the research and application of PGPM-*P. australis* combined remediation of heavy metal pollution in water.

## Materials and methods

### Plant pre-culture

The seeds of *P. australis* were collected from Lanzhou Botanical Garden, China (103^◦^42′16.99″ E, 36^◦^07′8.11″ N, 1583 m). Seeds were surface-sterilized with 2% (v/v) NaClO solution for 20 min, then washed with deionized water, and germinated on a petri dish (darkness at 25 ℃). After seed germination, the seedlings were sowed in 5 L plastic pots (43 × 19 cm, 12 seedings per pot) filled with water containing 1/4 Hoagland nutrient solution, which was sterilized at 121 ℃ for 2 h. The temperature throughout the growth process was maintained at 25 ± 1 ℃ on a 12 h light/12 h dark cycle, and the water was replenished every two days.

### Fungal strain culture

In this study, *T. citrinoviride* HT-1 (Accession number: MT781604.1) was preserved in the Microbial Collection of College of Life Science, Northwest Normal University. The fungal isolates were maintained on PDA medium (potato dextrose agar) at 4 ℃. The strain were resuscitated at room temperature for 1 h and then inoculated on PDA plates for 7 d under 28 °C 16 h light/8 h dark cycle. Conidial suspensions were harvested from the PDA mediums through using 2 mL sterile water, and then diluted to 10^7^ spore/mL for subsequent experiments [27].

### Experimental design

After 8 weeks of seed germination, *P. australis* plants with uniform growth were selected and placed in a 5 L plastic pot (36 plants per pot). A total of 30 pots were prepared for ten different treatments, each treatment contained three replicates: 0 mg/L Cd (sterile water); 0 mg/L Cd (sterile water) + strain HT-1; 5 mg/L Cd; 5 mg/L Cd + strain HT-1; 10 mg/L Cd; 10 mg/L Cd + strain HT-1; 15 mg/L Cd; 15 mg/L Cd + strain HT-1; 20 mg/L Cd; 20 mg/L Cd + strain HT-1; CdCl_2_ was used as the Cd source, which was added only once at the beginning of the treatment. After 24 h of Cd treatment, the strain HT-1 conidial suspension (10^7^ spore/mL) was inoculated into plants of the inoculation group at 5 mL/plant, the non-inoculation group was added with the same amount of sterile water. After 4 weeks, the plants were harvested for subsequent experiments.

### Cd uptake and translocation effect of *P. australis*

The treated *P. australis* were cleaned and divided into roots, stems and leaves for drying. 0.1 g of each plant sample was placed in a microwave digestion tube and treated with 5 mL nitric acid (HNO_3_) and 1 mL of 30% hydrogen peroxide (H_2_O_2_) until completely digested using microwave digestion instrument. The digestion liquid was filtered and transferred to a 25 mL volumetric flask and diluted to volume with 1% nitric acid. 50 mL hydroponic solution of each treatment group was filtered with 0.22 μm filter membrane. Cd concentrations were then determined with a flame atomic absorption spectrophotometer (AA7800, Shimadzu, Japan). Three biological replicates were randomly selected from each treatment.

The bioconcentration factor (BCF), translocation factor (TF) and Cd removal rate were calculated to determine the Cd bio-accumulation and the potential capacity of phytoremediation [28, 29].

$${\text{BCF}}=\frac{{\text{Cd concentration in }}\mathrm{ plant }}{\text{Cd concentration in }\mathrm{ water}}$$

$${\text{TF}}=\frac{{\text{Cd concentration in }}\mathrm{ shoot }}{\text{Cd concentration in }\mathrm{ root}}$$

$$\mathrm{Cd}\;\mathrm{removal}\;\mathrm{rate}\;\%=\frac{\left(\mathrm{Cd}\;\mathrm{initial}\;\mathrm{concentration}\;\mathrm{in}\;\mathrm{water}-\;\mathrm{Cd}\;\mathrm{concentration}\;\mathrm{after}\;\mathrm{plant}\;\mathrm{adsorption}\right)\;}{\mathrm{Cd}\;\mathrm{initial}\;\mathrm{concentration}\;\mathrm{in}\;\mathrm{water}}$$

### Growth index and physiological index of plants

The shoot length, root length, fresh weight of each sample were immediately measured after 28 days of exposure to Cd stress environment. Dried at 60 ^◦^C for a week, and weighed [30].

The roots of *P. australis* were cleaned 3–5 times by deionized water. Root morphological traits were scanned by a root scanner (V700 PHOTO, Epson, Japan), and WinRHIZO™2003b software (Regent Instruments, QC, Canada) was used to analyze total root surface area (SA) [31].

The root activity of *P. australis* was tested according to the triphenyl tetrazolium chloride (TTC) method [32]. A total of 0.1 g fresh *P. australis* roots were cut into pieces and immersed in 0.6% (w/v) TTC solution (TTC was dissolved in phosphate buffer at pH7.0) for 24 h at 30 ^◦^C in the dark. Then, the roots were rinsed twice, the water from the roots’ surfaces was removed, and the roots were immersed in 95% (v/v) ethanol for 30 min at room temperature. The absorbance values of the extraction solutions were tested by a spectrophotometer (Hitachi U3010, Tokyo, Japan) at 485 nm.

0.1 g leaves of the same part of each treated plant were collected. With 95% ethanol as solvent, grinded into homogenate on ice, filtered and diluted to 25 mL. The absorbance at 665, 649 and 470 nm were recorded (Hitachi U3010, Tokyo, Japan), with 95% ethanol as the blank, the whole experiment was carried out under dark conditions. The mean value was derived from three repeats. The chlorophyll content was tested by using the procedure of Li et al. [33].

The first fully expanded leaf at the top of the plant was used for measuring leaf gas exchange traits, including stomatal conductance (Gs), net photosynthesis rate (Pn), intercellular CO_2_ (Ci) and transpiration rate (Tr). A portable infrared gas exchange analyzer (GFS-3000, WALZ, Germany) was used, and measurements were taken between 9:00 h and 12:00 h Beijing time. The data were taken under 1200 μmol m^−2^ s^−2^ light intensity, 25 ^◦^C leaf temperature, and 440 μmol mol^−1^ CO_2_ concentration [33]. Leaves from six randomly selected seedlings from each treatment were clamped into the leaf chamber and measured after the net photosynthetic rate readings stabilized.

Malondialdehyde (MDA) was measured by the method of Mbonankira et al. [34]. Cold 10% trichloroacetic acid was added into a 0.1 g leaf sample and centrifuged at 4000 r/min for 10 min. Then, 2 mL of 0.6% thiobarbituric acid were added to the sample and incubated in a 100 ^◦^C water bath for 15 min. The supernatant was quickly cooled and centrifuged again. OD values were determined using an ELISA reader at 600 nm and 532 nm, respectively.

0.1 g leaf sample was ground in 1 mL extraction buffer (50 mM PBS, pH = 7.8), 1 mM EDTA-Na_2_, and 0.1% PVP) and centrifuged at 4 ^◦^C for 20 min at 12,000 rpm. The supernatant was used to determine the activities of superoxide dismutase (SOD), peroxidase (POD), catalase (CAT) and ascorbate peroxidase (APX). The activity of SOD was estimated using the method of Giannopolitis and Ries [35]. The activities of POD and CAT were assayed in accordance with Maehly and Chance’s [36]. The activity of APX was measured following the method of Nakano and Asada [37]. Each treatment was repeated at least three times [38].

### Transcriptome analysis

The *P. australis* root of 15 mg/L Cd + strain HT-1 treatment and only 15 mg/L Cd treatment were frozen in liquid nitrogen, and three biological replicates for transcriptomic analysis. Total RNA was extracted from samples using TRIzol reagent. (Invitrogen, Thermo Fisher Scientific Inc., Waltham, MA, USA). The quality of RNA was determined by using an Agilent 2100 Bioanalyzer (Agilent Technologies, Palo Alto, CA, USA) and a NanoDrop (Thermo Fisher Scientific Inc., Waltham, MA, USA). The RNA integrity numbers (RINs) of RNAs > 7 were selected to build the library and the cDNA library was sequenced on the Illumina sequencing platform by Personalbio Technology Co., Ltd. (Shanghai, China). The low-quality data and adaptor sequences in the original data were filtered out to ensure the accuracy of the data. De novo assembly was performed togenerate transcripts according to the Trinity method [39]. HTSeq (v0.6.1) was used to estimate the gene and isoform expression levels from paired-end clean data [40]. Differentially expressed genes (DEGs) were analyzed using DESeq2 (V1.6.3) in the Bioconductor software package [41]. Kyoto Encyclopedia of Genes and Genomes (KEGG) pathway enrichment analysis of DEGs was performed using KOBAS [42]. Gene Ontology (GO), an international standardized gene function classification, was performed using the BLAST2 GO tool [43].

### qRT–PCR analysis

An amount of 3 µg of purified total RNA was used as a template for first-strand cDNA synthesis using PrimeScript^TM^RT reagent Kit with gDNA Eraser (TaKaRa, Kyoto, Japan) for qPCR. Several genes identified by RNA-seq were selected for amplification using SYBR Green qPCR. Primers were designed using Sangon Biotech. Actin was used as a reliable internal reference gene for judging the efficiency of the RT-PCR system and the quality of RNA extracts (Table 1). qRT–PCR was conducted in a 10 μl volume containing. Six biological replicates were selected for measurement, the 2^−∆∆CT^ method was used to calculate relative expression levels [44, 45].

**Table 1:** qRT-PCR primers used in this study

Gene Name	Forward primer(5'-3')	Reverse primer(5'-3')
IAA	GGAGGTCTGTACGTGAAGGTGAG	GAGGAGAAGCAGCCGAAGAGC
ERF	ATGGATGCGACCTTCCGAGATTC ATGGATGCGACCTTCCGAGATTC	TCACCGAGTCACCCACAAACAC
APX	CGATGATGATGTCCACGCAGTTG	CCGATCCGATCCAGCATTCCAG
PCS	TTTACACTTCTTCTTGGCTGCTTGG	ACGCGACCTTGGCTATCAACTAC
MYB	CGAGGAGGATGCGAGACTGTTAG	GCCAAGTTGTTCACATAGCGATCTC
ATP	CTGCTTCTGGGTGCCCTTGG	ATCGTCGCCGTTGTCCGTATC
HMA	GCGGCAAGAGCGTTAATCACTG	AAGAGTTTCTGACCTTCCAGTAGCC AAGAGTTTCTGACCTTCCAGTAGCC
ABC	TACTGGTGCTGCTAAGATGGATGC	GGAGGATGTGCTCTCTGGTTTGG
ZIP	CACGCCGCAGCAAATCCAAG	CTCTCCAGGTTGAGCCGCTTG
WRKY	CTGGAGCAGCAGCAGCAGAC CTGGAGCAGCAGCAGCAGAC	GCGGCAGGAGCAAGGATGAC CACGCCGCAGCAAATCCAAG
Actin	CGAGCACGGTATTGTTAGCAACTG	CGCCTCAGTCAGCAGCACAG

### Statistical analysis

All data were analyzed by SPSS26.0 software for variance (one-way, ANOVA) and Duncan's multiple range test (*P* < 0.05). Columns were constructed by using Origin 2023 (Origin Software, Northampton, MA, USA).

## Results

### Effects of the growth of *P. australis* seedlings by the inoculation of strain HT-1 under Cd stress

**Fig. 1:**
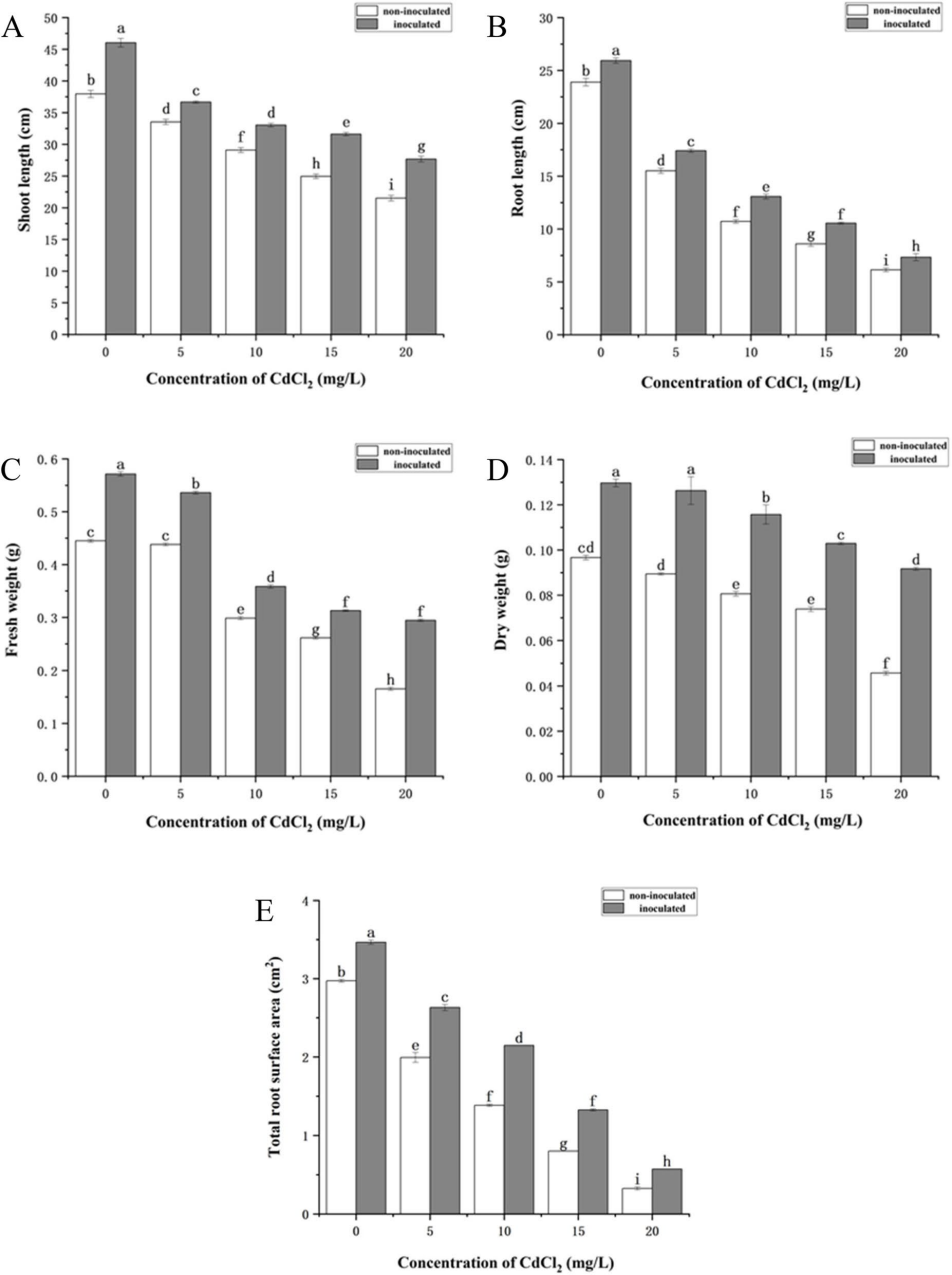
Effects of strain HT-1 on the growth of *P. australis* in different concentration of Cd, **A** shoot length, **B** root length, **C** fresh weight, **D** dry weight, **E** total root surface area. Values are mean ± SD (*n* = 15 plants). Different letters abovethe bars indicate the differences are significant at *P* < 0.05

**Fig. 2:**
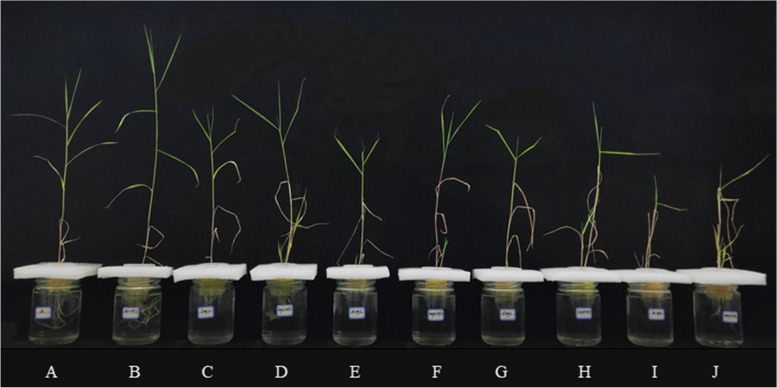
Effects of strain HT-1 on the growth of *P. australis* in different concentration of Cd, **A** 0 mg/L Cd (sterile water), **B** 0 mg/L Cd (sterile water) + strain HT-1, **C** 5 mg/L Cd, **D** 5 mg/L Cd + strain HT-1, **E** 10 mg/L Cd, **F** 10 mg/L Cd + strain HT-1, **G** 15 mg/L Cd, **H** 15 mg/L Cd + strain HT-1, **I** 20 mg/L Cd, **J** 20 mg/L Cd + strain HT-1

In the present study, the biomass of *P. australis* decreased with increasing CdCl_2_ concentration. However, strain HT-1 treatments significantly increased the shoot length, root length, fresh weight, dry weight and the total root surface area of plants (Figs. 1 and 2). Among them, after inoculation of strain HT-1, the increase of *P. australis* root length and the total root surface was the most significant under the concentration of CdCl_2_ was 15 mg/L, the growth rates were 22.79% and 65.44% respectively compared with the non-inoculated plants (Fig. 1A, E). The increase of the shoot length, fresh weight and dry weight of *P. australis* were the most significant under the the concentration of CdCl_2_ was 20 mg/L, which were increased by 28.62%, 78.05% and 100.66% respectively compared with the non-inoculated plants (Fig. 1B, C, D).

### Effects of Cd accumulation and transport in *P. australis* by the inoculation of strain HT-1

We measured Cd of the enrichment factor (BCF), transport factor (TF) and plant removal rate. As shown in Table 2, When the concentration of CdCl_2_ was 20 mg/L, the content of Cd^2+^ was the highest in leaf, stem, root, which were 15.29 mg/kg, 16.36 mg/kg and 73.24 mg/kg, respectively. The strain HT-1 significantly promoted the absorption Cd^2+^ of *P. australis*. Under different Cd treatment concentrations, the Cd^2+^ content in roots, stems and leaves of inoculated was significantly higher than that of non-inoculated plants. When the concentration of CdCl_2_ was 5 mg/L, the Cd^2+^ content in the leaves and stems of the inoculated increased most significantly, and the growth rates were 408.10% and 235.54% compared with the non-inoculated treatment, respectively. When the concentration of CdCl_2_ was 15 mg/L, the Cd^2+^ content of roots increased most significantly with the inoculation of strain HT-1, the growth rate was 20.13% compared with the non-inoculated treatment.

**Table 2:** The effect of strain HT-1 inoculation on Cd uptake and transport in *P. australis*

Cd treatment (mg/L)		Leaf Cd content (mg/kg)	Stem Cd content (mg/kg)	Root Cd content (mg/kg)	BCF	TF	Cd removal rate (%)
0	non-inoculated	ND	ND	ND	ND	ND	ND
	inoculated	ND	ND	ND	ND	ND	ND
5	non-inoculated	3.62 ± 0.01 h	7.57 ± 0.11 h	65.23 ± 0.54 h	15.28 ± 0.09 b	0.17 ± 0.0029 h	74.44 ± 0.13 e
	inoculated	18.39 ± 0.04 d	25.40 ± 0.21 d	71.85 ± 0.57 e	23.13 ± 0.15 a	0.61 ± 0.0028 d	87.32 ± 0.97 d
10	non-inoculated	8.90 ± 0.02 g	12.13 ± 0.32 g	68.40 ± 0.12 g	8.94 ± 0.05 e	0.31 ± 0.0077 g	87.13 ± 0.31 d
	inoculated	19.10 ± 0.06 c	38.40 ± 0.33 a	76.95 ± 0.28 c	13.44 ± 0.03 c	0.75 ± 0.0117 c	88.44 ± 0.01 d
15	non-inoculated	9.88 ± 0.02 f	14.79 ± 0.02 f	70.01 ± 0.18 f	6.31 ± 0.01 g	0.35 ± 0.0008 f	90.83 ± 0.25 c
	inoculated	38.74 ± 0.03 a	37.22 ± 0.03 b	84.10 ± 0.07 a	10.67 ± 0.01 d	0.90 ± 0.0019 a	96.56 ± 0.04 a
20	non-inoculated	15.29 ± 0.17 e	16.36 ± 0.18 e	73.24 ± 0.13 d	5.24 ± 0.02 h	0.43 ± 0.0060 e	92.87 ± 0.14 b
	inoculated	36.90 ± 0.06 b	31.52 ± 0.03 c	82.83 ± 0.15 b	7.56 ± 0.00 f	0.83 ± 0.0043 b	97.63 ± 0.04 a

Different lowercase letters in the same column indicated significant difference between treatments (*P* < 0.05)

As showed in Table 2, it can be seen that the BCF of *P. australis* decreases with the increase of CdCl_2_ concentration in water, indicating that *P. australis* have better enrichment ability in low Cd environment. After inoculation with strain HT-1, the BCF of Cd in *P. australis* increased significantly. The maximum BCF was observed in *P. australis* at 15 mg/L CdCl_2_, which was increased by 69.10% compared with the non-inoculated treatment. At the same time, the TF of *P. australis* increased with the increase of CdCl_2_ concentration in water, indicating that with the transport capacity of plants to heavy metals gradually increased with the increase of its concentration, which led to more heavy metals transported from roots to shoots, and was beneficial to plants to absorb more heavy metals. After inoculated strain HT-1, the transport coefficient of plants in each treatment group was significantly higher than that of non-inoculated, among them, TF increased most significantly when the concentration of CdCl_2_ was 5 mg/L, and the growth rate was 258.82% compared with non-inoculated plants. In addition, with the increase of CdCl_2_ concentration, the removal rate of Cd in water by *P. australis* showed an increasing trend, and the removal rate of Cd by plants was significantly increased after inoculation with strain HT-1. When CdCl_2_ was 5 mg/L, the inoculation of strain HT-1 had the most significant effect on the Cd removal rate of *P. australis*. Compared with the non-inoculated treatment, the growth rate was 17.3%.

### Physiological response of strain HT-1 to *P. australis* seedlings under Cd stress

**Fig. 3:**
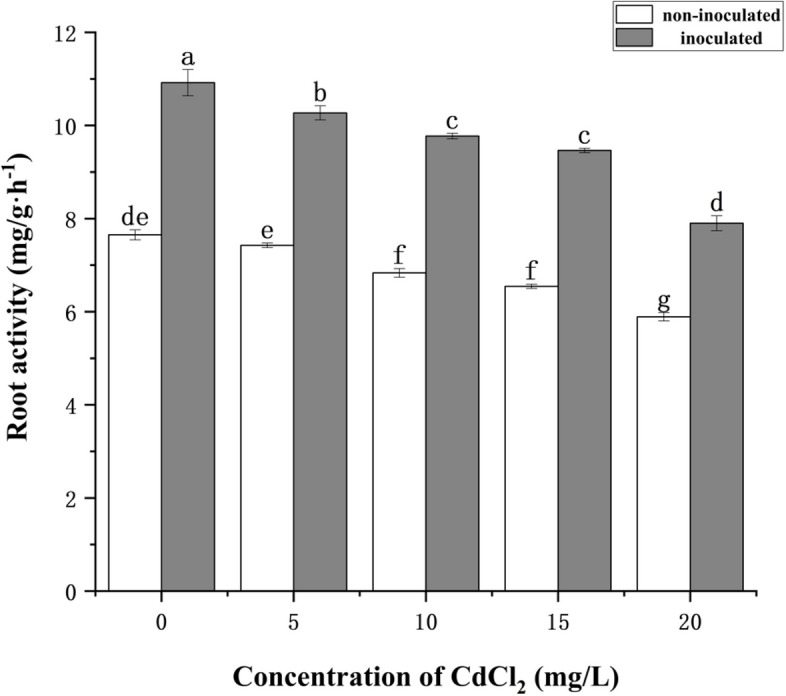
Effects of strain HT-1 on root activity of *P. australis* in different concentration of Cd. Values are mean ± SD (*n* = 15 plants). Different letters abovethe bars indicate the differences are significant at *P* < 0.05

As shown in Fig. 3, with the increase of CdCl_2_ concentration, the root activity decreased significantly. Strain HT-1 treatments significantly increased the root activity of *P. australis*. At the concentration of CdCl_2_ of 15 mg/L, the root activity of inoculated with strain HT-1 had the highest growth rate of 44.57%. The difference was significant at the 0.05 level (*P* < 0.05).

**Fig. 4:**
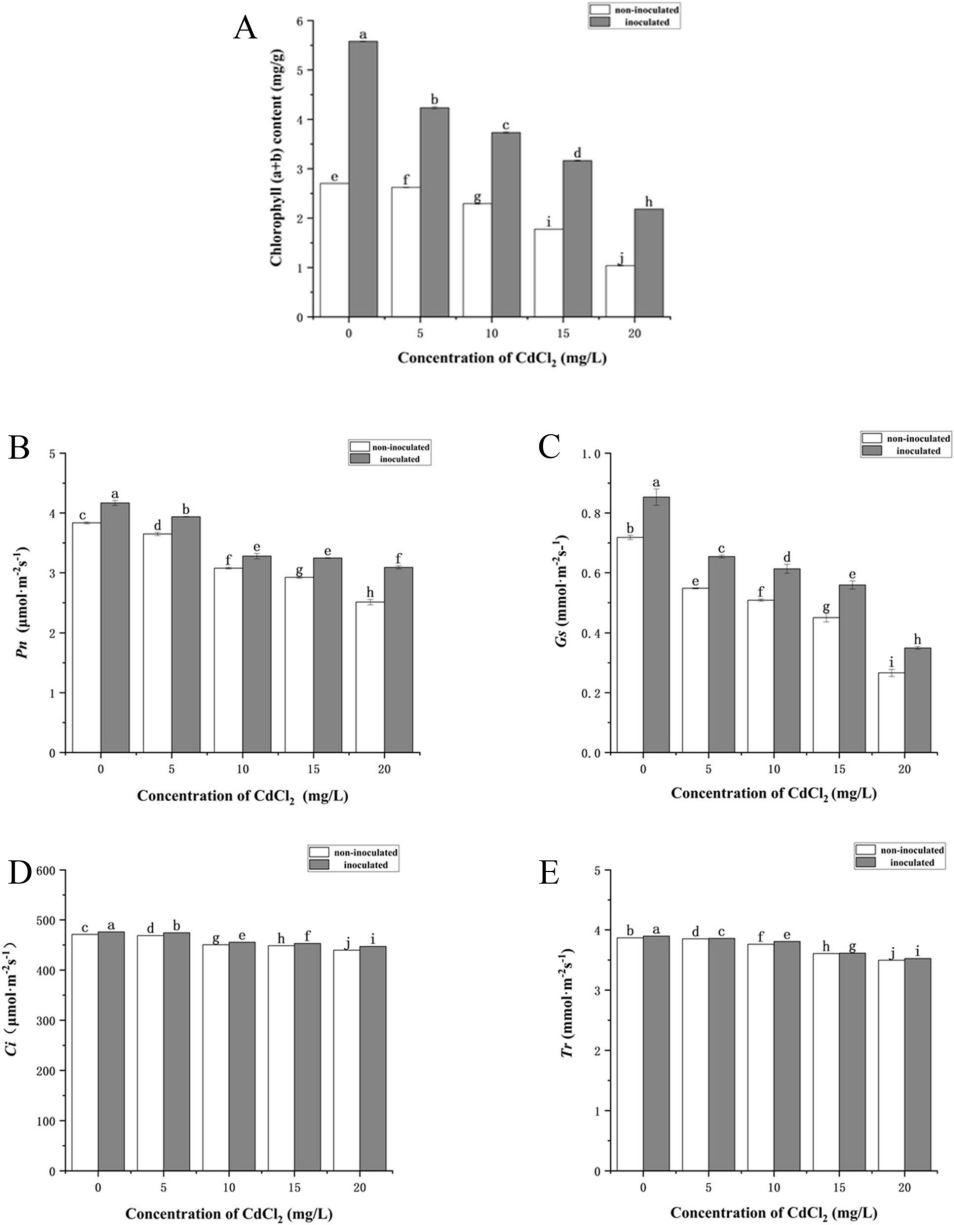
Effects of strain HT-1 on the photosynthesis of *P. australis* in different concentration of Cd, **A** chlorophyll content, **B** net photosynthetic rate (Pn), **C** leaf stomatal conductance (Gs), **D** intercellular CO_2_ concentration (Ci), **E** transpiration rate (Tr). Values are mean ± SD (*n* = 15 plants). Different letters abovethe bars indicate the differences are significant at *p* < 0.05

In this study, we measured the changes of photosynthetic pigment contents in *P. australis* (Fig. 4). With the increase of CdCl_2_ concentration, the chlorophyll content (a + b) of plants decreased significantly. Strain HT-1 treatments significantly increased the chlorophyll content of *P. australis*. Among them, after inoculation of strain HT-1, the increase of chlorophyll content was the most significant under the concentration of 20 mg/L CdCl_2_, the growth rate was 110.25% compared with the non-inoculated plants (Fig. 4A). In addition, with the increase of CdCl_2_ concentration, the leaf gas exchange attributes in *P. australis*, including net photosynthetic rate (Pn), leaf stomatal conductance (Gs), intercellular CO_2_ concentration (Ci) and transpiration rate (Tr) decreased significantly. However, strain HT-1 inoculation significantly increased Pn, Gs, Ci and Tr of *P. australis* under CdCl_2_ treatment (*P* < 0.05) (Fig. 4B, C, D, E). These results indicate that strain HT-1 protects the *P. australis* photosynthesis under heavy metal Cd stress.

**Fig. 5:**
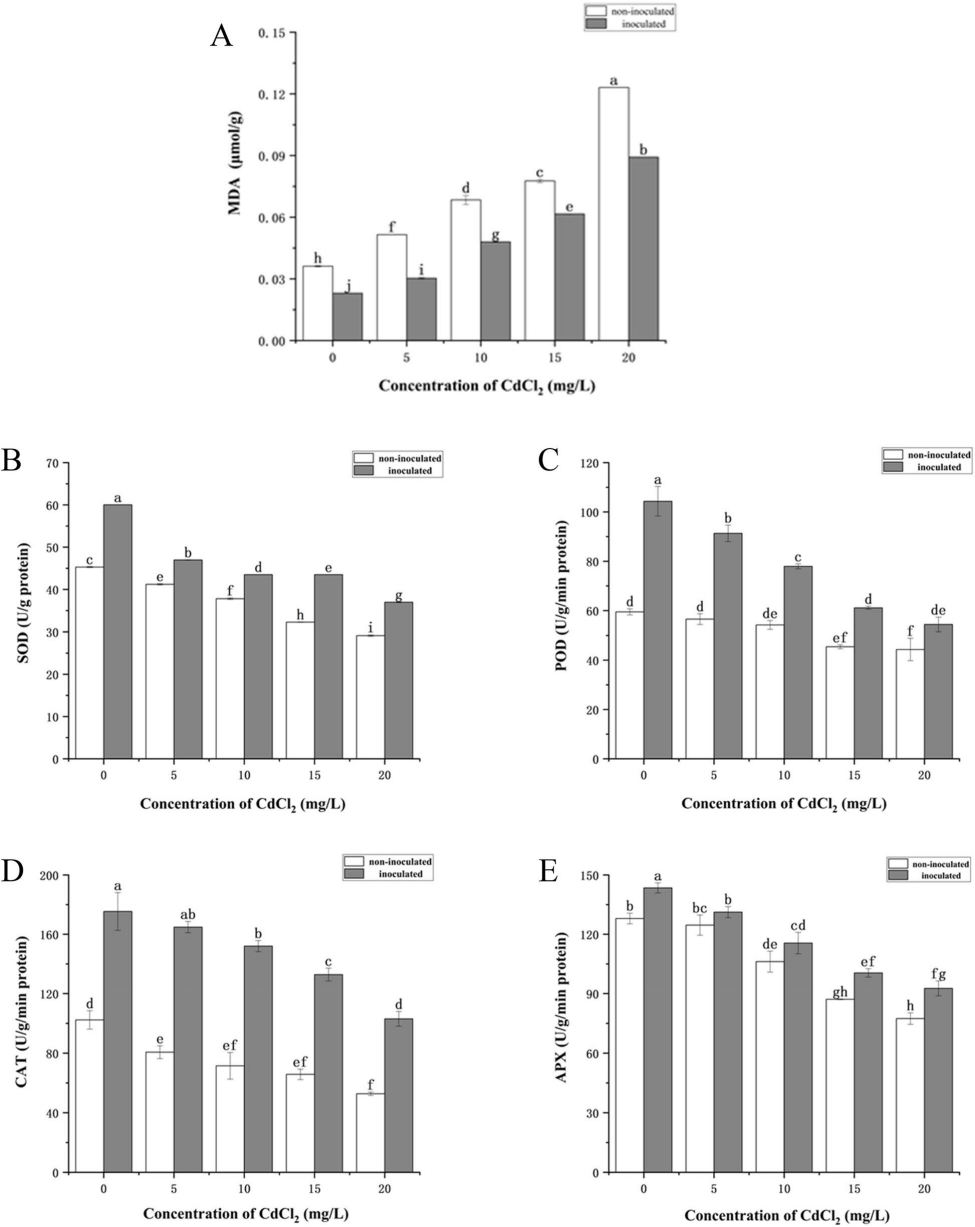
Effects of strain HT-1 on the antioxidant system of *P. australis* in different concentration of Cd, **A** MDA content, **B** SOD content, **C** POD content, **D** CAT content, **E** APX content. Values are mean ± SD (*n* = 15 plants). Different letters abovethe bars indicate the differences are significant at *p* < 0.05

It can be seen that activities of antioxidant enzyme (SOD, POD, CAT and APX) in *P. australis* leaves were significantly lower than control seedlings with the increase of CdCl_2_ concentration (Fig. 5B, C, D, E). After inoculation with strain HT-1, the activities of SOD, POD, CAT and APX at most were 0.33, 0.75, 1.13 and 0.20 times higher than those of uninoculated plants, respectively, which were significantly increased the activities of antioxidant enzymes (*P* < 0.05). In addition, we found that the content of MDA in *P. australis* leaves increased significantly with the increase of CdCl_2_ concentration. However, strain HT-1 treatments significantly redused the MDA content of *P. australis*. Among them, after inoculation of strain HT-1, the reduction of MDA content was the most significant under the concentration of CdCl_2_ was 5 mg/L, the reduction rate was 41.10% compared with the non-inoculated plants (Fig. 5A).

In summary, heavy metal cadmium seriously damaged the structure and function of *P. australis* leaves and root tissues. Strain HT-1 can alleviate the damage of Cd to plant photosynthetic system, improve the antioxidant capacity of plants, and thus improve the tolerance of plants to heavy metals. Combined with physiological and biochemical analys8is, it can be found that when the concentration of CdCl_2_ is 15 mg/L, the inoculation strain HT-1 has the most significant improvement in the indicators of *P. australis* seedlings. At this time, the strain exerts its best biological function. Therefore, we next performed transcriptome analysis on the roots of *P. australis* seedlings treated with 15 mg/L CdCl_2_ to explore the molecular mechanism of strain HT-1 improving *P. australis* to alleviate Cd toxicity in polluted environment.

### Unigene annotation and identification of DEGs

**Fig. 6:**
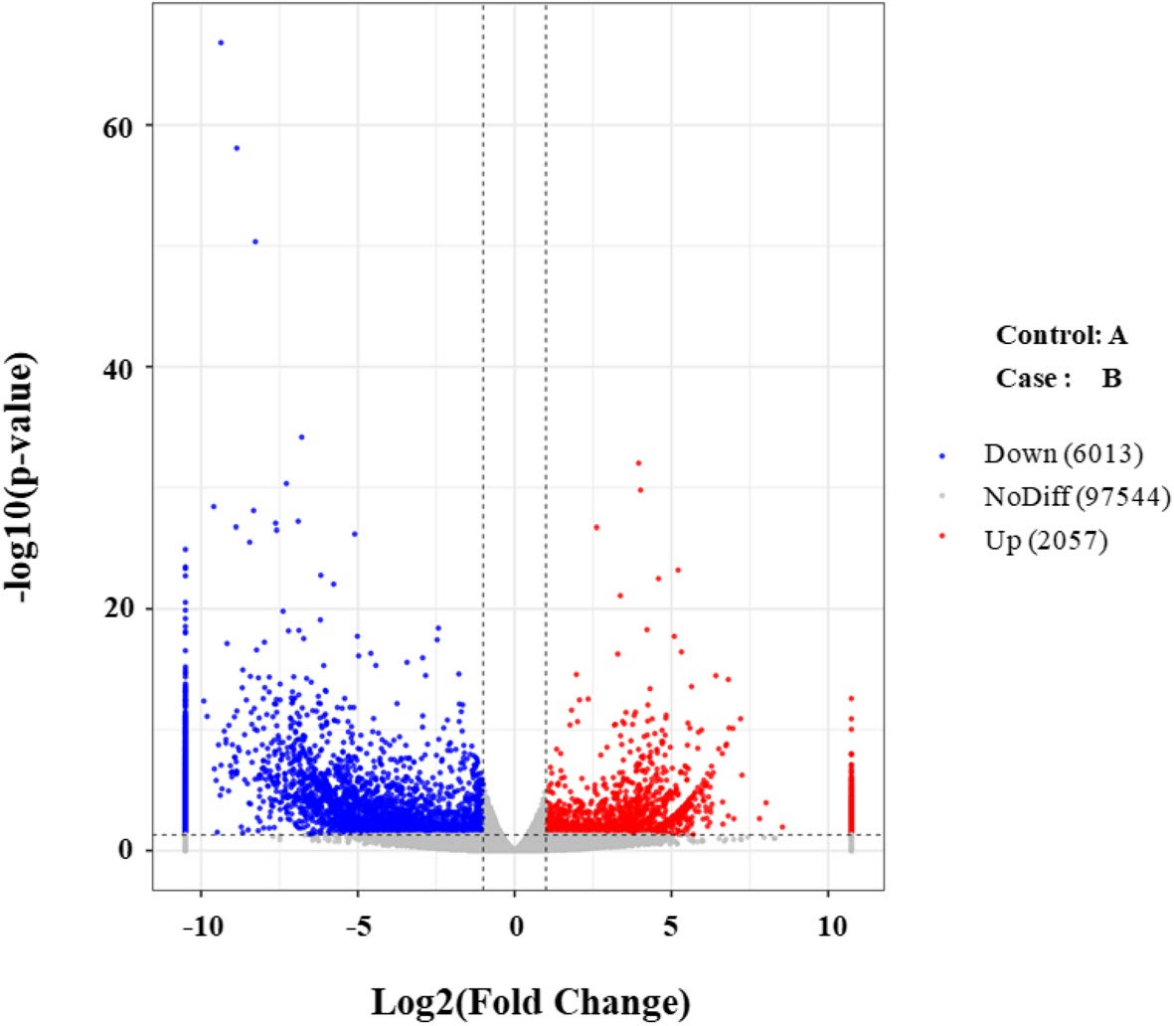
Volcano plots of differentially expressed genes, **A** 15 mg/L Cd treatment, **B** 15 mg/L Cd + strain HT-1 treatment

A total of 118,927,346 and 131,503,608 raw reads were obtained from the inoculation treatment and non-inoculation treatment groups, respectively. 117,143,178 (inoculation treatment) and 129,347,506 (non-inoculation treatment) clean reads were retained after assembly (Table 3). Sequencing data were submitted to the NCBI (https://www.ncbi.nlm.nih.gov/), accession number was PRJNA1032252. A total of 518,426 unique sequences were annotated based on blastx alignment searches of six public databases including GO, KEGG, NR, eggNOG, Swiss-prot and Pfam. A total of 8070 genes were identified, of which 2057 genes were up-regulated (**|**logFC**|**> 1) and 6013 genes (**|**logFC**|**> 1) were down-regulated (Fig. 6).

**Table 3:** Summary of trimming and read mapping results of the sequences obtained from *P. australis* roots treated without or with strain HT-1 inoculation

Sample	Raw_reads	Clean_reads
15 mg/L Cd (1)	40,758,436	40,177,876
15 mg/L Cd (2)	41,123,416	40,477,238
15 mg/L Cd (3)	37,045,494	36,488,064
15 mg/L Cd + strain HT-1 (1)	44,118,746	43,369,004
15 mg/L Cd + strain HT-1 (2)	39,453,560	38,754,802
15 mg/L Cd + strain HT-1 (3)	47,931,302	47,223,700

### GO function and KEGG pathway enrichment analysis of the DEGs

**Fig. 7:**
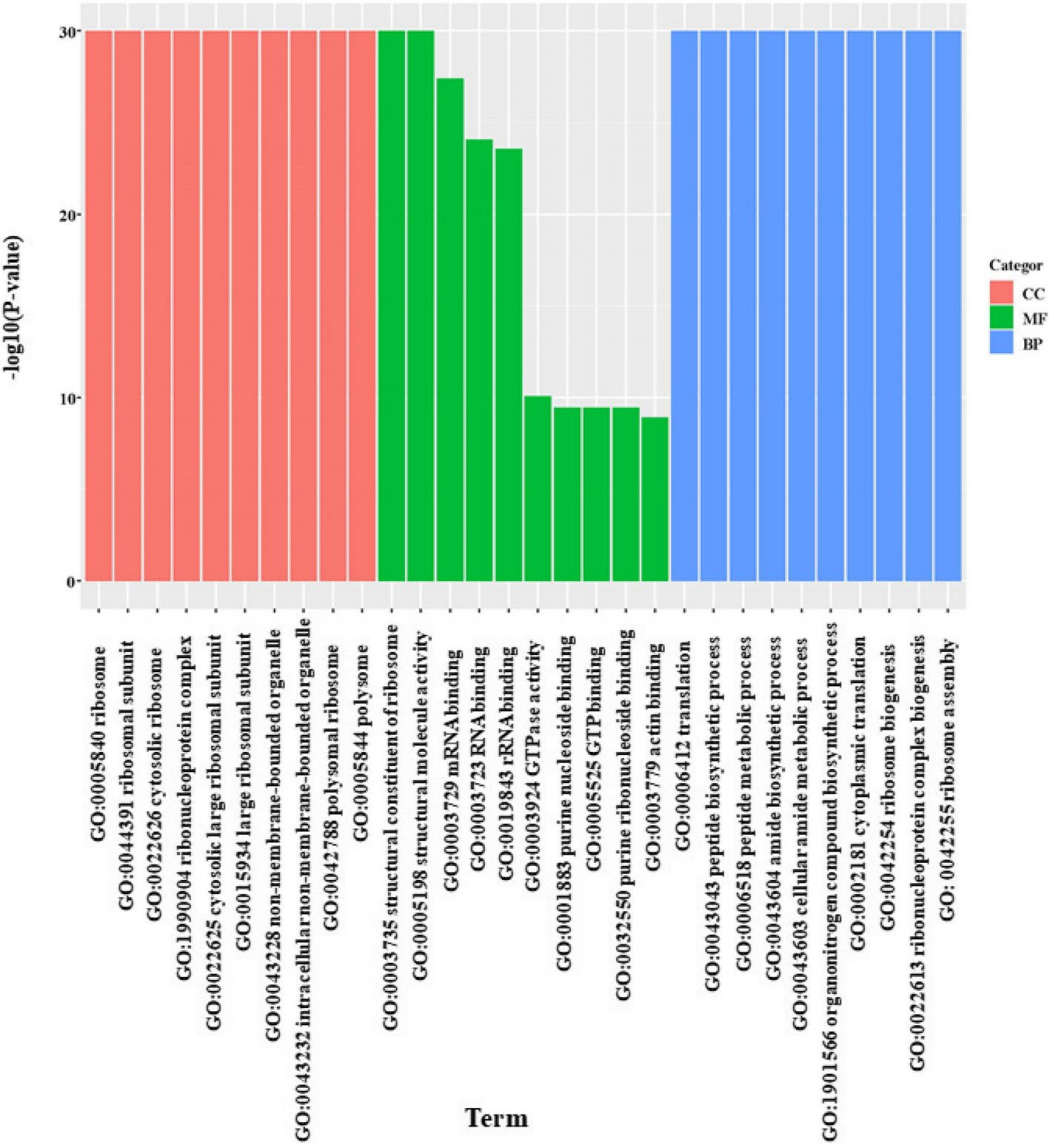
Chart summarizing the results of Gene Ontology (GO) enrichment analysis

GO analysis elucidated the specific biological functions of DEGs in the two comparisons,which were classified in three ontologies (biological processes (BP), molecular functions (MF) and cellular components (CC). The top 10 significantly enriched GO terms concerning were shown in (Fig. 7). The upregulated DEGs were mainly involved in catalytic activity, structural constituent of the ribosome (MF), cellular nitrogen compound biosynthetic process, peptide metabolic process and translation (BP), extracellular region and ribosome (CC). The downregulated DEGs were significantly enriched in strutural molecule activity (MF), ribonucleoprotein complex, non-membrane-bounded organelle and ribosome (CC), and translation (BP). GO analysis indicated that strain HT-1 could effectively enhance such as the catalytic activity, ribosome metabolism, translation, etc., and defense mechanism of plants. So as to improve the Cd tolerance and enable plant survival better in stressed environments.

**Fig. 8:**
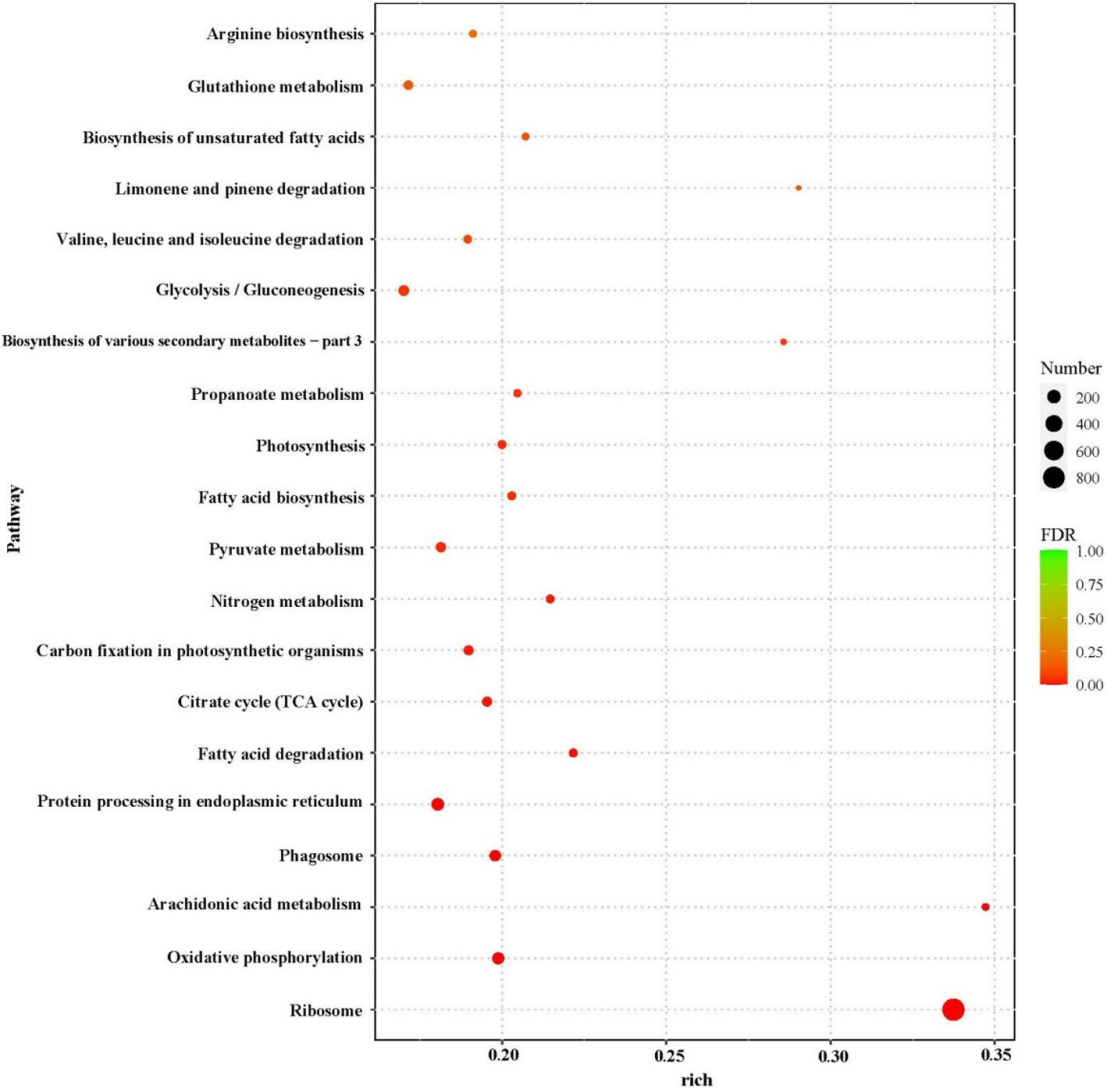
Kyoto Encyclopedia of Genes and Genomes (KEGG) enrichment analysis of differentially expressed genes (DEGs)

All DEGs were assigned to the KEGG database for KEGG pathway enrichment analysis. Research found that DEGs were significantly enrich in metabolic pathways and biosynthesis of secondary metabolites. KEGG enrichment analysis as scatter plots with the 20 most significantly enriched pathways. Of which, the ribosome, endocytosis, limonene and pinene degradation, protein processing in endoplasmic reticulum and photosynthesis were the top five enrich pathways in upregulated DEGs (Fig. 8). In contrast, the ribosome, oxidative phosphorylation, arachidonic acid metabolism, citrate cycle and nitrogen metabolism were the top five pathways in downregulated DEGs. KEGG analysis indicated that strain HT-1 can regulated amino acid metabolism, nitrogen metabolism, carbohydrate metabolism and strengthen signal transduction to reduce the toxicity of Cd to plants.

### Transcription factors (TF) analysis

**Fig. 9:**
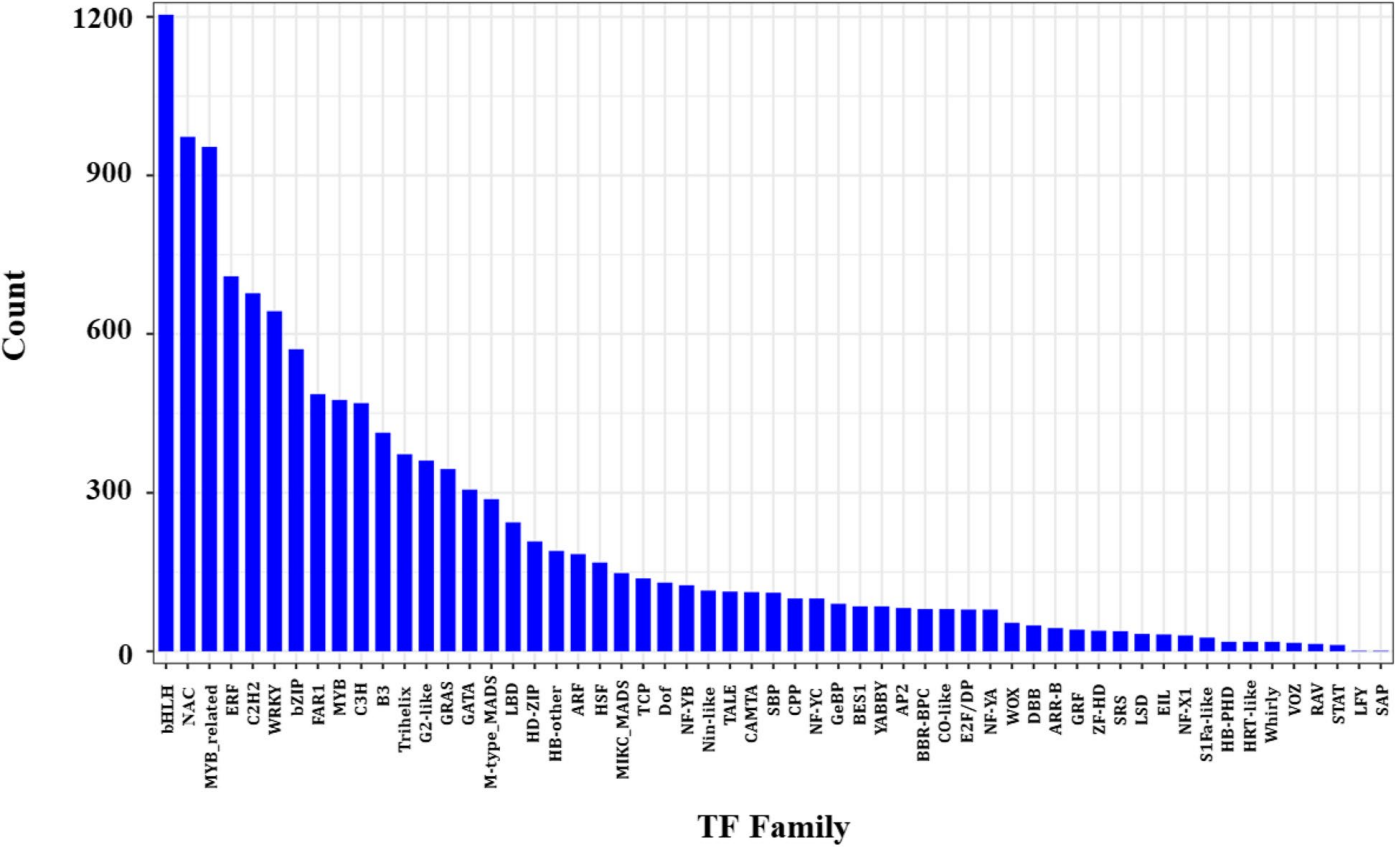
Changes in gene expression

Changes in gene expression caused by strain HT-1 ultimately regulate the response mechanism of *P. australis* roots to Cd stress. This research examined 12,577 hypothetical TFs from 57 different families. The top 10 TFs were bHLH (1204), NAC (973), MYB-related (954), ERF (709), C2H2 (677), WRKY (643), bZIP (571), FAR1 (486), MYB (475), C3H (469) (Fig. 9).

### Verification of partial DEGs using RT-qPCR

**Fig. 10:**
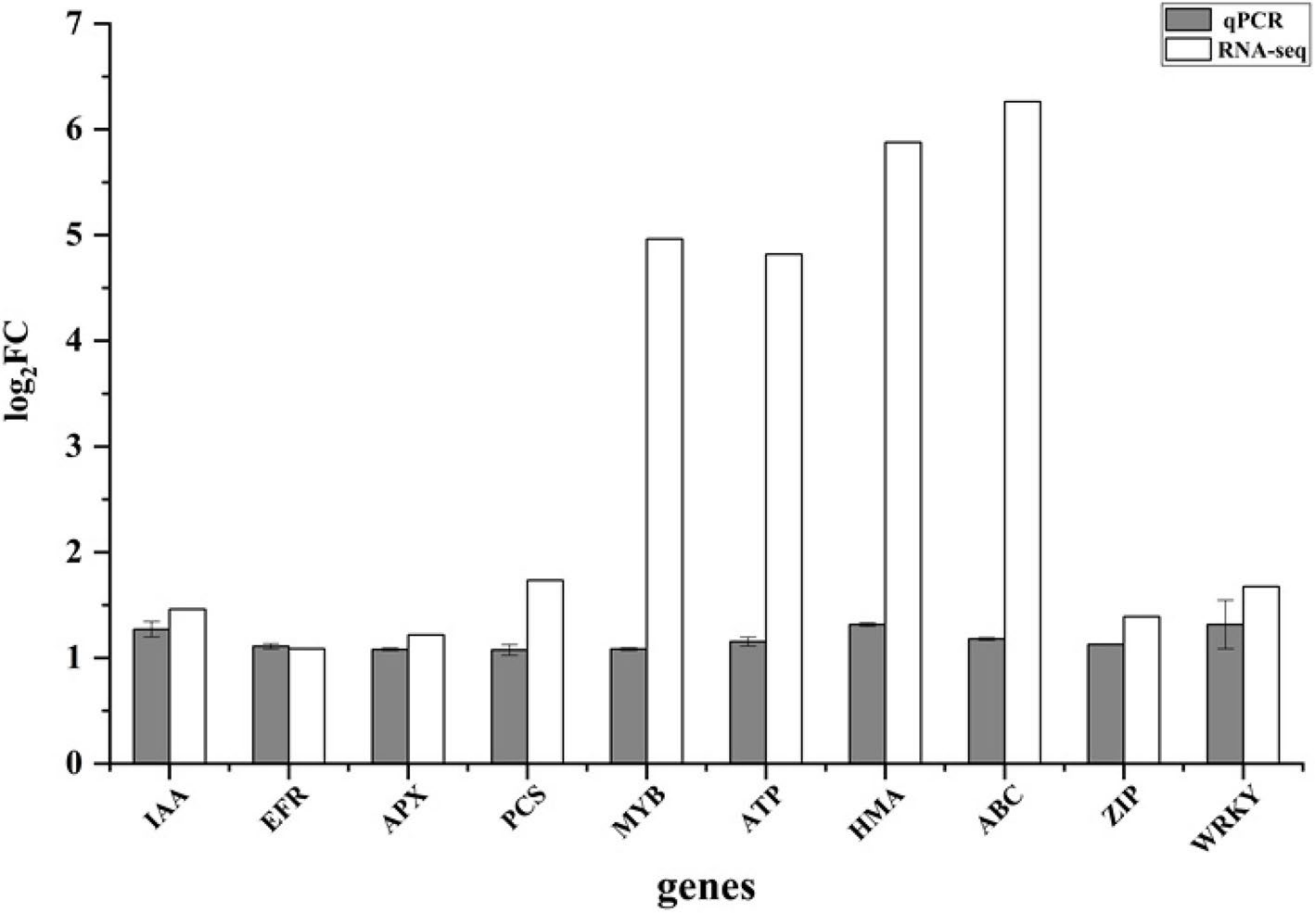
qRT-PCR assay of genes in *P. australis*

To validate the reliability of RNA-Seq data, we selected 10 DEGs related to plant growth, photosynthesis, antioxidant activities and metal transport, including IAA (DN1943_c1_g1), ERF (DN7252_c0_g1), APX (DN1099_c0_g1), PCS (DN1373_c0_g2), MYB (DN29668_c0_g1), ATP (DN88929_c1_g1), HMA (DN139448_c0_g1), ABC (DN38393_c0_g1), ZIP (DN19222_c0_g1) and WRKY (DN1823_c1_g1) (Table 4), quantitative analysis of gene expression was detected using real-time quantitative PCR (qPCR) examined them by qRT-PCR. The primers used for qRT-PCR are listed in Table 4. As expected, the expression profiles of these DEGs were consistent with the RNA-seq results (Fig. 10), indicating the dependability of the RNA-Seq data.

**Table 4:** The annotation of selected functional genes

Gene name	Gene ID	NR Annotation
IAA	DN1943_c1_g1	Auxin-responsive protein
ERF	DN7252_c0_g1	hylene-responsive transcription factor
APX	DN1099_c0_g1	ascorbate peroxidase
PCS	DN1373_c0_g2	Photosystem II protein
MYB	DN29668_c0_g1	Transcription factor MYB3R-5
ATP	DN88929_c1_g1	Plasma membrane ATPase
HMA	DN139448_c0_g1	Cadmium/zinc-transporting ATPase
ABC	DN38393_c0_g1	ABC transporter family
ZIP	DN19222_c0_g1	homeobox-leucine zipper protein
WRKY	DN1823_c1_g1	WRKY transcription factor

## Discussion

As a non-essential element, the accumulation of trace amounts of Cd in plants can result in dwarfing of plants, yellowing of leaves, slowed root growth, and hindered overall plant growth [46, 47]. Previous studies has shown that PGPM provides sufficient nutrients for plants by producing siderophores, solubilizing P and fixing nitrogen, and can also synthesize plant growth-regulating hormones to promote plant growth and enhance resistance in heavy metal-contaminated environments [48, 49]. In our study, with the increase of CdCl_2_ treatment concentration, the growth of plants was significantly inhibited. Plant growth was significantly improved after treatment with strain HT-1. As a plant growth-promoting bacteria, strain HT-1 has the function of producing IAA and siderophores, as well as antibacterial and disease resistance [28, 50]. Therefore, after inoculated strain HT-1, it can directly lead to an increase in *P. australis* biomass and indirectly promote plant growth by increasing plant resistance to heavy metals.

The efficiency of remediation depends on the bioactivity of residual heavy metals in the environment and their availability in plants [51, 52]. Apart from plant growth promotion, PGPM were proven can also assist plant heavy metal uptakes and accumulation via increasing their solubility and bioavailability [53]. Wang et al. resulted that PGPM inoculants enhanced oilseed Cd concentration and Cd phytoextraction efficiency, and the soil Cd removal rate of PGPM inoculated plants was 2.44 times higher than that of non-inoculated plants [54]. Asilian et al. inoculated *Piriformospora indica* increased plant root Cd concentration and the uptake of maize [55]. Chen et al. reported that inoculating *Sphingomonas* SaMR12 significantly increased Zn uptake by *S. alfredii* was close to 23 fold [56]. In this study, inoculating strain HT-1 significantly improved the absorption and transportation of Cd by *P. australis*, and enhanced the removal rate of Cd in water. Our results show that inoculation with strain HT-1 can effectively improve the remediation efficiency of *P. australis* to Cd in water.

Plant growth depends on the cycle between the above-ground and below-ground parts, with the above-ground part synthesizes the products of photosynthesis into a carbon source and the below-ground part continuously absorbs and transports water and nutrients [46]. Therefore, the degree of root development and the strength of photosynthesis can be used as a basis for evaluating plant growth status [57]. In our study, plant total root surface area and root activity, the chlorophyll content, net photosynthetic rate, stomatal conductance, intercellular CO_2_ concentration and transpiration rate of *P. australis* leaf showed that the inoculated treatment was better than non-inoculated treatments. It indicates that strain HT-1 improves the coordination between photosynthesis and root growth of *P. australis*, thus promoting the physiological metabolism of plants.

Heavy metal Cd is an inducer of oxidative stress, which typically leads to lipid peroxidation of plant cell membranes and generates a significant amount of reactive oxygen species (ROS), resulting in oxidative damage to plants [52, 58]. In addition, Cd toxicity also inhibits the photoactivation of photosystem II (PSII), which leads to the destruction of chloroplasts in leaves and indirectly promotes the production of ROS [59, 60]. Unregulated ROS in plant cells can disrupt cell integrity and function, leading to detrimental effects on plant growth [61]. Therefore, under cadmium stress, plants activate their own antioxidant defense system and increase the activities of antioxidant enzymes such as SOD, POD, APX, CAT and GR to scavenge toxic free radicals and protect themselves from oxidative stress [62, 63]. In this study, with the increase of CdCl_2_ treatment concentration, the activities of antioxidant enzymes in *P. australis* decreased significantly. However, the inoculated strain HT-1 could significantly increase the activities of four antioxidant enzymes, thereby helping plants to alleviate Cd stress, which was consistent with the results of Pan et al. and Raja et al. [64, 65], indicating that PGPM could change enzyme activity and increase the ability of plants to resist oxidative stress. The generation of ROS and the subsequent oxidative stress resulted in high MDA content. MDA is a byproduct of lipid peroxidation that usually used to assess the extent of cell membrane damage [66]. High levels of MDA can lead to a decrease in cell water content and membrane integrity which negatively affects plant metabolic functions, yield and growth [67]. In our study, Regardless of whether Cd stress occurred or not, inoculation with strain HT-1 significantly reduced the content of MDA in *P.australis* leaves. This reduction was significantly different from the non-inoculated treatments. It indicated that strain HT-1 could alleviate damage to the plant cell membrane system and improve the tolerance of *P.australis* to Cd. The result is consistent with the research of Wang et al. [68].

Cd stress can induce the expression of stress-related genes and proteins [69]. The effect of strain HT-1 also alters the expression of plant genes and proteins [28]. In previous studies, RNA-seq sequencing has been extensively utilized to investigate the gene and protein responses of gramineous plants, including rice [70], wheat [31], and maize [71] under heavy metal stress. However, RNA-Seq analysis of *P. australis* under Cd stress is limited. In this study, 8070 differentially expressed genes were mainly involved in ribosome, amino acid metabolism and other pathways. Therefore, we studied the DEGs from plant hormone signal transduction, oxidative phosphorylation and plant-microorganism interaction. Indoleacetic acid (IAA) promoted plant fixation of mineral elements, which played a positive role in plant growth [72]. As a main transcription factor protein, ERF potentially involved in the growth process of various plants [73]. APX, a DEG associated with antioxidant enzymes, plays an important r ole in plant antioxidant defense system [31]. As a subunit of photosystem II (PSII), PCS participates in plant photosynthesis-related physiological activities, and its expression level is positively correlated with plant photosynthesis capacity [74]. In our study, after inoculation with strain HT-1, the expression levels of the above genes in *P. australis* were significantly increased. This is similar to the findings of Liu et al. who observed an increase in the expression of IAA and CAT genes in plants after treating Cd-stressed wheat with silicon [31]. The results show that strain HT-1 can mediate the transduction of signaling pathways, such as plant hormone synthesis and antioxidant defense system. It can also promote the expression of related genes, thereby alleviating oxidative stress and enhancing plant tolerance to cadmium. In addition, this study also screened a large number of DEGs related to Cd uptake and transport, including zinc ion transmembrane transporter, ABC transporter and metal ion transmembrane transporter, as well as key Cd transporters such as MYB, ATP and HMA. ZIP family transporters are mainly present in the inner membrane system of plants and play an indispensable role as Zn/Cd transporters [75]. ABC transporters can be strictly involved in plant metal transport [76–79]. In this study, the treatment with strain HT-1 significantly affected the expression of these genes, indicating that strain HT-1 enhanced the repair capacity of *P. australis* for Cd by regulating the transporters of heavy metals. Khan et al. also reported that the expression of a large number of genes in the HMA family was significantly affected after melatonin treatment of Cd-stressed cotton seedlings [80]. This suggests that HMA may play a role in melatonin-induced relief of Cd stress. In summary, the results of this study indicate that strain HT-1 activates transcription factors through signal transduction, which in turn triggers the expression of a series of functional genes. This activation reduces the growth inhibition of *P. australis* caused by Cd and ultimately enhances the plants' tolerance to Cd. The ability to absorb and transport Cd has been greatly improved.

## Conclusion

In this study, the effects of plant endophytic growth-promoting microorganisms on Cd tolerance and repair ability of *P. australis* were analyzed by physiological, biochemical and transcriptomic methods. We found that strain HT-1 induced significant up-regulated the expression of genes related to oxidative phosphorylation and ribosome pathways, thereby increasing the growth rate, photosynthetic rate, antioxidant capacity and Cd uptake and transport rate of *P.australis*, enhancing *P. australis* remediation efficiency and resistance to Cd stress. This study provides new insights for PGPM to improve plant tolerance to heavy metals and remediation efficiency.

## Data Availability

No datasets were generated or analysed during the current study.
